# Association of triglyceride-glucose index with risk of cardiovascular disease among patients with prediabetes: population based prospective cohort study

**DOI:** 10.1186/s12872-025-05112-w

**Published:** 2025-09-29

**Authors:** Man Li, Hong-Peng Zhang, Yan-Mei Cheng, Li-Xia Sun, Shuo-Hua Chen, Xiang-Jie Duan, Chun Xian, Shou-Ling Wu, Hai-Yan Yin

**Affiliations:** 1 Department of Intensive Care Unit, The First Affiliated Hospital of Jinan University, 613 Huangpu Ave W, Guangzhou, 510630 China; 2 The First Affiliated Hospital of Jinan University, Guangzhou, China; 3 Department of Cardiothoracic surgery ICU, The First Affiliated Hospital of Sun Yat-sen University, Guangzhou, China; 4 Department of Emergency, The Affiliated Hospital of North China University of Science and Technology, Tangshan, China; 5 Department of Cardiology, Kailuan Hospital, Tangshan, China

**Keywords:** Prediabetes, Triglyceride glucose index, Cardiovascular disease, Cohort study, Insulin resistance

## Abstract

**Background:**

The triglyceride-glucose (TyG) index was associated with an increased risk of cardiovascular disease (CVD) in patients with prediabetes. However, this association has not yet been evaluated in prospective studies. Hence, we aimed to investigate the association between the TyG index and the risk of incident CVD in patients with prediabetes.

**Methods:**

Participants with prediabetes were enrolled from the Kailuan study and were followed up for clinical events until 2022. The exposure variable was TyG index. The primary outcomes were the incidence of CVD events, including ischemic heart disease and stroke, while the secondary outcomes were each specific type of CVD events. Cox proportional hazards regression models and restricted cubic spline analysis were used to calculate multivariable-adjusted hazard ratios and investigate exposure-response association, respectively.

**Results:**

Over a mean follow-up period of 14.19 years, 13.81% (*n* = 2,537) of the 18,364 participants with prediabetes developed CVD events. Compared with participants in the lowest TyG index quartile (Q1, TyG index < 8.45), those in Q2 (TyG index 8.45–8.81), Q3 (TyG index 8.81–9.23), and Q4 (TyG index ≥ 9.23) were associated with an increased risk of CVD, with hazard ratios (HRs) of 1.18 (95% confidence interval [CI] 1.05–1.34), 1.29 (95% CI 1.15–1.46), and 1.45 (95% CI 1.28–1.63), respectively. For each unit increase in TyG index, the risk of CVD events increased by 12% in prediabetes patients. We also found a linear association between baseline TyG index and CVD events in prediabetes patients (*P*-non-linear = 0.12).

**Conclusions:**

TyG index is an independent risk factor for incident CVD in the prediabetes patients.

**Supplementary Information:**

The online version contains supplementary material available at 10.1186/s12872-025-05112-w.

## Introduction

Diabetes mellitus is a metabolic disorder associated with numerous serious comorbidities and is the ninth leading cause of death worldwide [1, 2]. Prediabetes, an intermediate stage between normal glucose homeostasis and diabetes mellitus, is rapidly increasing in prevalence worldwide, especially in China [3]. The prevalence of prediabetes among adults in China was reported to be 15.5% in 2007 and 38.1% in 2018 [4, 5], indicating that at least one-third of Chinese adults are now living with prediabetes. Cardiovascular disease (CVD) is the primary cause of mortality in the world and accounts for more than 40% of deaths in China [6, 7]. Like diabetes, prediabetes is also associated with multiple comorbidities, including CVD [8–10]. AusDiab study showed patients with prediabetes, as defined by impaired fasting glucose, had a 2.5-fold (1.2–5.1) higher relative risk of CVD morbidity and mortality than normal glucose tolerance [9, 11]. Thus, it is crucial for patients with prediabetes to identify variable CVD risk factors and implement early interventions to prevent disease onset.

Insulin resistance, indicated by a reduced tissue response to insulin, is a critical mechanism in diabetes mellitus that begins as early as the prediabetes stage [12–14]. It affects not only glucose metabolism but also lipid metabolism, thereby promoting the development of CVD [15]. Although the euglycemic-hyperinsulinemic clamp is considered as the gold standard for identifying insulin resistance, this method is too cumbersome for large epidemiological studies [16]. Recently, the triglyceride-glucose (TyG) index has been widely used to measure insulin resistance due to its noninvasiveness, simplicity, and convenience [17]. Over the past decade, epidemiological evidence has linked the TyG index with CVD in both diabetes patients and the general population [18–21]. However, data on the association between the TyG index and CVD in prediabetes patients are relatively limited. A recent study found a linear, positive association between the TyG index and the risk of CVD among 2,370 prediabetes patients aged 65 years or younger [22]. This research, however, was based on a cross-sectional study of young and middle-aged participants, suggesting only a short-term role of the TyG index in increasing CVD risk during the prediabetes stage. There remains a lack of longitudinal studies tracking patients with prediabetes over time to understand the interconnections between early TyG index levels and the development of CVD events. Therefore, using data from the Kailuan study, an ongoing, prospective, community-based cohort, we investigated the long-term association of the TyG index with the risk of incident CVD and its subtypes among prediabetes patients.

## Method

### Data source

This longitudinal cohort study used data from the Kailuan Study (Clinical trial number: ChiCTR-TNC-11001489), which is an ongoing, large, prospective cohort study with follow-up assessments conducted every two years. Details of Kailuan study have been described elsewhere [23, 24]. In brief, 101,510 individuals were recruited between 2006 and 2007 (referred to as “2006”) in Tangshan, Hebei Province, China. Participants completed structured questionnaires during the interviews and underwent standardized clinical examinations, including measurements of fasting blood glucose (FBG) and triglycerides (TG). Detailed information on the structured questionnaires and clinical examinations can be found in previously published literature by our research group [25]. These questionnaires and examinations were repeated at each biennial follow-up visit, with the most recent round conducted in 2022–2023. Furthermore, annual surveillance was conducted to assess the incidence of CVD and all-cause mortality through linkage with hospital discharge records and medical insurance databases. The study complied with the Helsinki Declaration and was approved by the Kailuan General Hospital Ethics Committee, China (2006–05). All participants provided written informed consent prior to inclusion in the study.

### Study participants

Patients with prediabetes who participated in baseline examination (2006–2007) and had complete data on FBG and TG were recruited. Prediabetes was defined according to the 2024 ADA criteria as an FBG level of 5.6–6.9 mmol/L, with no use of hypoglycemic medications, based on data collected during the baseline examination (2006–2007) [26]. Patients with pre-existing CVD, heart failure, or cancer were excluded from the study.

### Exposure and outcomes

On the morning of the health examination, a fasting venous blood sample (8–12 h) of 5 ml was collected from each participant. FBG and TG levels were determined using the hexokinase/glucose-6-phosphate dehydrogenase method and the enzymatic colorimetric method, respectively, with an autoanalyzer (Hitachi Automatic Analyzer 7600, Tokyo, Japan). The TyG index was calculated as ln [fasting TG (mg/dL) × FBG (mg/dL)/2] based on data collected during the baseline examination (2006–2007) [27].

The primary outcome of this analysis was the incidence of CVD events, a composite of ischemic heart disease and stroke, with each outcome further analyzed separately as secondary outcomes. Stroke included both ischemic and hemorrhagic stroke; hemorrhagic stroke encompassed subarachnoid hemorrhage and intracerebral hemorrhage. Ischemic heart disease included myocardial infarction and revascularization therapy. Revascularization therapies consisted of procedures such as coronary artery bypass grafting (CABG) and percutaneous coronary intervention (PCI). Myocardial infarction and stroke were identified by ICD-10 codes, CABG and PCI were identified using the International Classification of Diseases, Ninth Revision, Clinical Modification (ICD-9-CM) codes [28]. Information on cardiovascular events was collected annually from discharge summaries and medical insurance databases of Kailuan Group-affiliated hospitals and designated municipal hospitals. All potential cases were initially identified using ICD codes and were subsequently verified by trained medical personnel through review of hospitalization records. Final confirmation was performed by a panel of senior physicians based on comprehensive clinical documentation. Participants were followed from baseline until the first occurrence of a CVD event, death, or the end of follow-up on December 31, 2022, whichever came first.

### Data collection

Demographic data (e.g., age and sex), socioeconomic status (e.g., education), lifestyle information (e.g., drinking status, smoking status, and physical activity), medication use (e.g., antihypertensive, hypoglycemic, and lipid-lowering drugs), family history (e.g., CVD) and medical history (e.g., cancer, heart failure, and CVD) were obtained through face-to-face interviews using a standardized questionnaire. The detailed measurement procedures have been described previously [25]. Anthropometric parameters and blood pressure were measured during biennial health examinations. Body mass index (BMI) was calculated by dividing body weight (kg) by the square of height (m [2]) [29]. Hypertension was defined as systolic blood pressure ≥ 140 mmHg and/or diastolic blood pressure ≥ 90 mmHg or having a history of hypertension or using antihypertensive drugs. Physical activity was defined as the frequency of physical activity during leisure time (≥ 30 min per session, ≥ 3 times per week). Current smokers were defined as individuals who smoked at least one cigarette per day in the past year. Current drinkers were defined as the consumption of ≥ 2 standard drinking volume/d for men and ≥ 1 standard drinking volume/d for women (1 standard drinking volume is equivalent to 120 ml of wine, 360 ml of beer or 45 ml of liquor) in the past year [30].

During the health examination, blood samples were collected from the antecubital vein after a fasting period of at least 8 h overnight. Biochemical parameters, including TG, low-density lipoprotein cholesterol (LDL-C), high-density lipoprotein cholesterol (HDL-C), total cholesterol (TC), and FBG levels, were measured using an autoanalyzer (Hitachi Automatic Analyzer 7600, Tokyo, Japan). High-sensitivity C-reactive protein (hs-CRP) levels were assessed with an autoanalyzer (Cias Latex CRP-H, Kanto Chemical, Tokyo, Japan).

### Statistical analysis

Baseline characteristics were described as the mean (SD), median with IQR, or number and percentage (%), as appropriate. Intergroup comparisons across TyG index quartiles were conducted by one-way analysis of variance (ANOVAs) or Kruskal–Wallis tests for continuous variables and χ [2] test for categorical variables. We used multiple imputation by chained equations to handle missing covariate data.

Unadjusted incidence rates (per 1,000 person-years) and Kaplan‒Meier curves were used to present the absolute risk of CVD events. Cox proportional hazards models were used to analyze the hazard ratios (HRs) and 95% confidence intervals (CIs) for the risk of incident CVD associated with the TyG index, both as a continuous variable and in quartile categories. Model 1 was adjusted for age (continuous variable, years) and sex (men or women). Model 2 adjusted for variables in model 1 and current smoking (yes or no), current drinking (yes or no), physical activity (active or inactive), education (high school completion and lower or college completion and higher), snoring (never or ever) and CVD family history (yes or no). Model 3 adjusted for variables in model 2 and BMI (continuous variable, kg/m^2^), TC (continuous variable, mmol/L), LDL-C (continuous variable, mmol/L), HDL-C (continuous variable, mmol/L), log-transformed (hs-CRP) (continuous, mg/L), hypertension (yes or no), antihypertensive (yes or no) and lipid-lowering drugs (yes or no). Furthermore, we calculated the population-attributable risk (PAR) to theoretically estimate the association between TyG index and CVD in prediabetes patients. To further explore the association between TyG index and risk of CVD events, restricted cubic splines with three knots at the 25th, 50th, and 75th percentiles were used. In these spline models, the covariates included in Model 3 were adjusted.

### Subgroup analyses

We performed subgroup analyses to investigate the association between the TyG index and CVD events in different subgroups. Patients with prediabetes were classified based on the following characteristics: age (< 45, 45–60, and ≥ 60 years), sex (men and women), current smoking (no and yes), current drinking (no and yes), snoring (never and ever), BMI (< 24 kg/m^2^, 24–28 kg/m^2^, and ≥ 28 kg/m^2^), and hs-CRP (≤ 3 mg/L and > 3 mg/L). We tested for interactions by including multiplicative interactions between the TyG index quartiles and the stratified factors in Model 3 to assess their impact on CVD outcomes.

### Sensitivity analyses

To assess the robustness of the association between TyG index and CVD in patients with prediabetes, several sensitivity analyses were performed. To begin with, to address potential reverse causation, we excluded participants who experienced incident CVD within the first year of follow-up. Second, to minimize the impact of drugs on our findings, we excluded participants who were using antihypertensive or lipid-lowering drugs at baseline. Third, we assessed the impact of multiple imputation on results by re-analyzing the model using the complete dataset without imputation. Fourth, to account for the potential impact of unmeasured confounders, we determined the E-value needed to quantify their potential effect on the results using the adjusted Model 3 [31]. Fifth, to address the competing risk of death, we used the Fine-Gray model to analyze the difference in the risk of CVD events between different TyG index groups, controlling for the competing risk of death. Sixth, to evaluate whether the association between TyG index and CVD is stronger when using more recent metabolic data, and to assess the temporal consistency of the association, we used TyG index data from the most recent clinical examination before the end of follow-up as the exposure variable. Seventh, to examine whether baseline TyG index remains an independent predictor of CVD after accounting for recent metabolic changes, we further adjusted for the most recent TyG index in the baseline model. Eighth, to assess whether the observed associations were primarily driven by ischemic rather than hemorrhagic events, we excluded participants who developed hemorrhagic stroke during follow-up. Finally, to evaluate whether the observed association was confounded by subsequent diabetes onset, we excluded participants who developed diabetes during follow-up.

### Prediction model

To evaluate the predictive performance of TyG index for CVD, the C-index, Net Reclassification Index (NRI) and Integrated Discrimination Improvement Index (IDI) of the Prediction Model for Atherosclerotic Cardiovascular Disease Risk in China (China-PAR model), China-PAR model plus TG, China-PAR model plus FBG and China-PAR model plus TyG index were calculated to compare the predictive performance of different indicators for CVD.

### Secondary outcome analyses

Cox proportional hazards models were applied to evaluate the associations between the TyG index and the risk of incident ischemic heart disease, stroke, ischemic stroke, and hemorrhagic stroke, with adjustments for the same covariates as previously described. In addition, restricted cubic splines were employed to further investigate potential dose-response association.

Statistical analyses were performed using SAS software (version 9.4; SAS Institute, Cary, NC, USA) and R software (version 4.1.2; R Foundation for Statistical Computing, Vienna, Austria). A two-tailed *P*-value of < 0.05 was considered statistically significant, except for interaction testing, where a *P*-value of < 0.10 was considered significant [32, 33].

## Results

### Patient selection

**Fig. 1:**
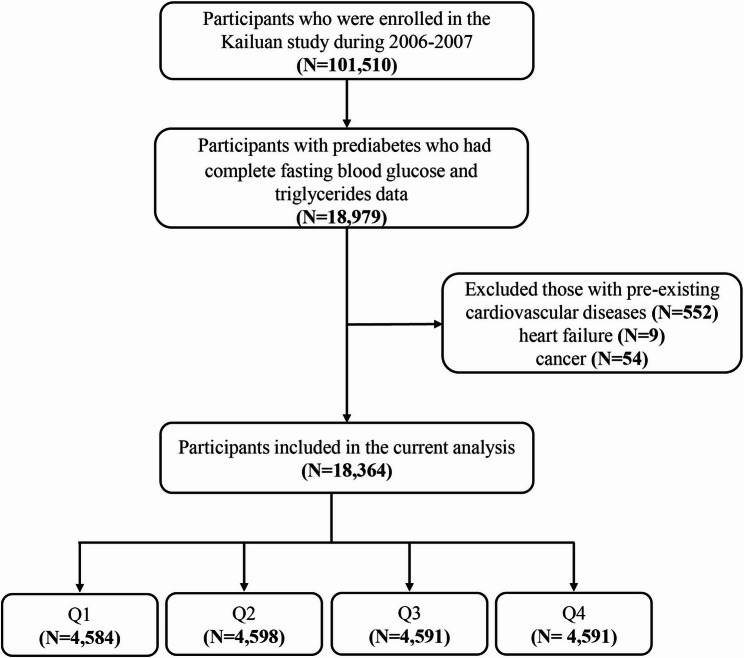
Flow chart of study participants

Figure 1 shows the process of participant selection. We included 18,979 prediabetes patients who had complete data on FBG and TG. After excluding participants with pre-existing CVD (*N* = 552), heart failure (*N* = 9) or cancer (*N* = 54), 18,364 patients were included in the final cohort.

### Cohort characteristics

Table 1 shows the baseline characteristics of participants overall and according to TyG index quartiles. Individuals with higher TyG index levels were generally younger, more likely to be current smokers, drinkers, or snorers, and exhibited higher levels of FBG, TG, BMI, systolic blood pressure, and hs-CRP, but lower LDL levels. In addition, they were more likely to use antihypertensive and lipid-lowering drugs.

**Table 1:** Baseline characteristics of the study population

	Total (*N* = 18364)	Q1 (*N* = 4584)	Q2 (*N* = 4598)	Q3 (*N* = 4591)	Q4 (*N* = 4591)
TyG index^a^	8.87 ± 0.62	8.15 ± 0.24	8.63 ± 0.10	9.00 ± 0.12	9.71 ± 0.41
TyG index^b^	8.93 ± 0.69	8.53 ± 0.58	8.77 ± 0.56	9.02 ± 0.58	9.40 ± 0.72
Age, year	52.15 ± 11.56	52.00 ± 12.18	52.50 ± 11.78	52.82 ± 11.44	51.27 ± 10.73
Male, n (%)	15,709 (85.54)	3889 (84.84)	3933 (85.54)	3901 (84.97)	3986 (86.82)
Fasting blood glucose, mmol/L	6.06 ± 0.33	5.97 ± 0.30	6.04 ± 0.32	6.09 ± 0.34	6.12 ± 0.36
Triglyceride, mmol/L	1.38 (0.98–2.11)	0.77 (0.64–0.87)	1.17 (1.07–1.27)	1.67 (1.51–1.86)	3.05 (2.46–4.31)
Systolic blood pressure, mmHg	133.91 ± 20.97	129.54 ± 20.26	133.20 ± 20.95	135.35 ± 21.07	137.56 ± 20.78
Total cholesterol, mmol/L	5.08 ± 1.13	4.85 ± 0.97	5.04 ± 0.97	5.25 ± 1.02	5.19 ± 1.45
hs-CRP, mg/L	0.90 (0.34–2.20)	0.71 (0.30–1.97)	0.80 (0.31-2.00)	0.95 (0.39–2.50)	1.03 (0.43–2.62)
Body mass index, kg/m^2^	25.58 ± 3.45	24.18 ± 3.26	25.21 ± 3.22	26.16 ± 3.40	26.77 ± 3.37
Low density lipoprotein cholesterol, mmol/L	2.52 ± 0.87	2.52 ± 0.88	2.56 ± 0.79	2.55 ± 0.86	2.43 ± 0.93
High density lipoprotein cholesterol, mmol/L	1.53 ± 0.38	1.54 ± 0.38	1.54 ± 0.37	1.51 ± 0.37	1.53 ± 0.41
Higher education, n (%)	1935 (10.54)	515 (11.23)	443 (9.63)	475 (10.35)	502 (10.93)
Current smoking, n (%)	6914 (37.65)	1751 (38.20)	1613 (35.08)	1715 (37.36)	1835 (39.97)
Current drinking, n (%)	7817 (42.57)	2004 (43.72)	1778 (38.67)	1941 (42.28)	2094 (45.61)
Physical activity, n (%)	2946 (16.04)	792 (17.28)	662 (14.40)	806 (17.56)	686 (14.94)
Snoring, n (%)	7208 (39.25)	1832 (39.97)	1636 (35.58)	1859 (40.49)	1881 (40.97)
Hypertension, n (%)	9178 (49.98)	1757 (38.33)	2259 (49.13)	2482 (54.06)	2680 (58.38)
CVD family history, n (%)	2227 (12.13)	560 (12.22)	529 (11.51)	570 (12.42)	568 (12.37)
Antihypertensive drugs, n (%)	2032 (11.07)	351 (7.66)	459 (9.98)	570 (12.42)	652 (14.20)
Lipid-lowering drugs, n (%)	123 (0.67)	28 (0.61)	26 (0.57)	31 (0.68)	38 (0.83)

Q1: TyG index^a^ <8.45, Q2: TyG index^a^ 8.45–8.81, Q3: TyG index^a^ 8.81–9.23, Q4: TyG index^a^ ≥9.23

*Abbreviation: TyG* triglyceride glucose, *hs-CRP* high-sensitivity C-reactive protein, *CVD* cardiovascular diseases

^a^: data from the baseline examination

^b^: data from the most recent clinical examination before the end of follow-up

### Associations of TyG index with CVD in patients with prediabetes

**Fig. 2:**
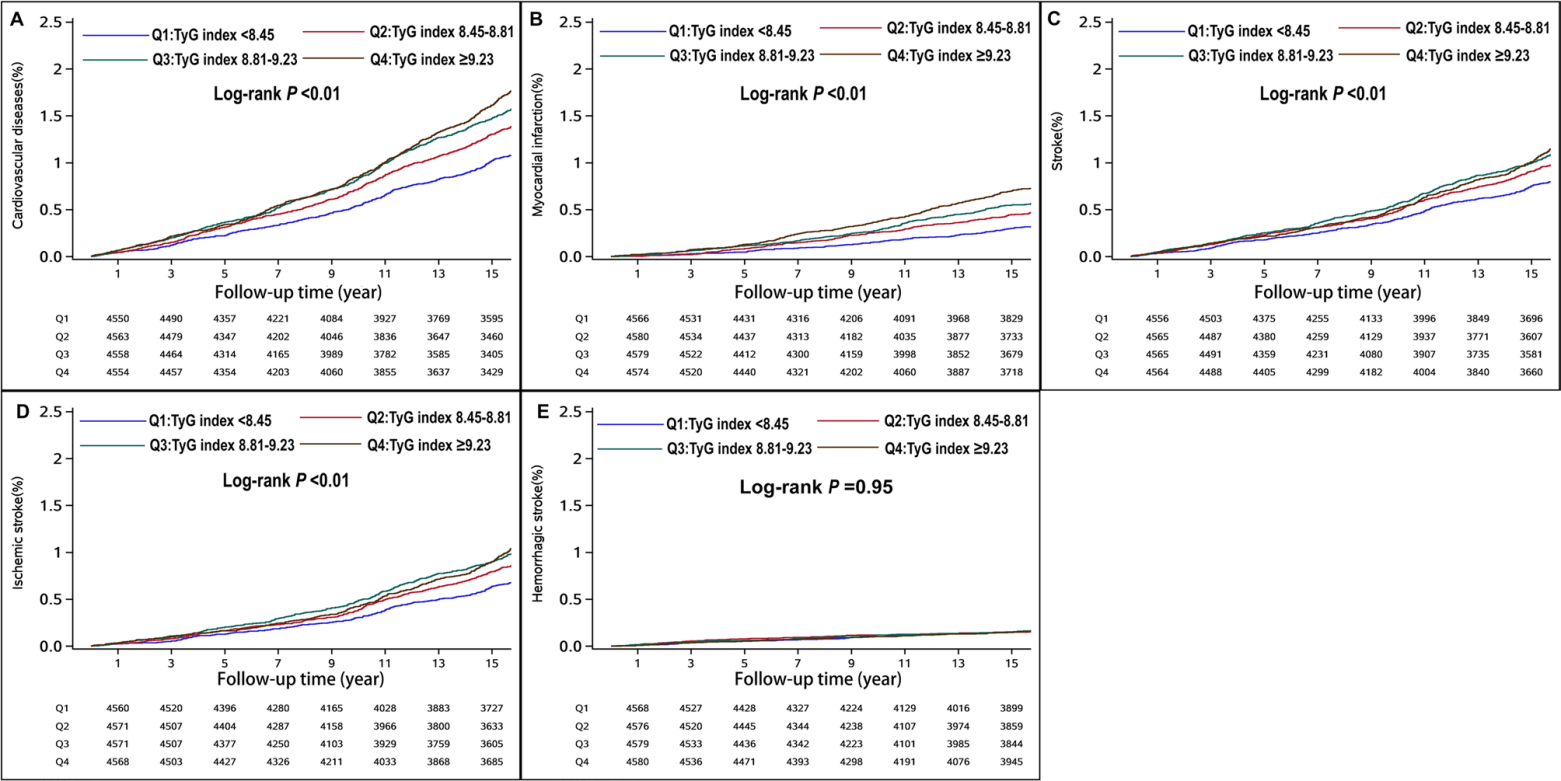
Cumulative incidence of cardiovascular diseases and its subtypes according to categories of TyG index in prediabetes patients Panel **A**: Cardiovascular diseases; Panel **B**: Ischemic heart disease; Panel **C**: Stroke; Panel **D**: Ischemic stroke; Panel **E**: Hemorrhagic stroke

**Fig. 3:**
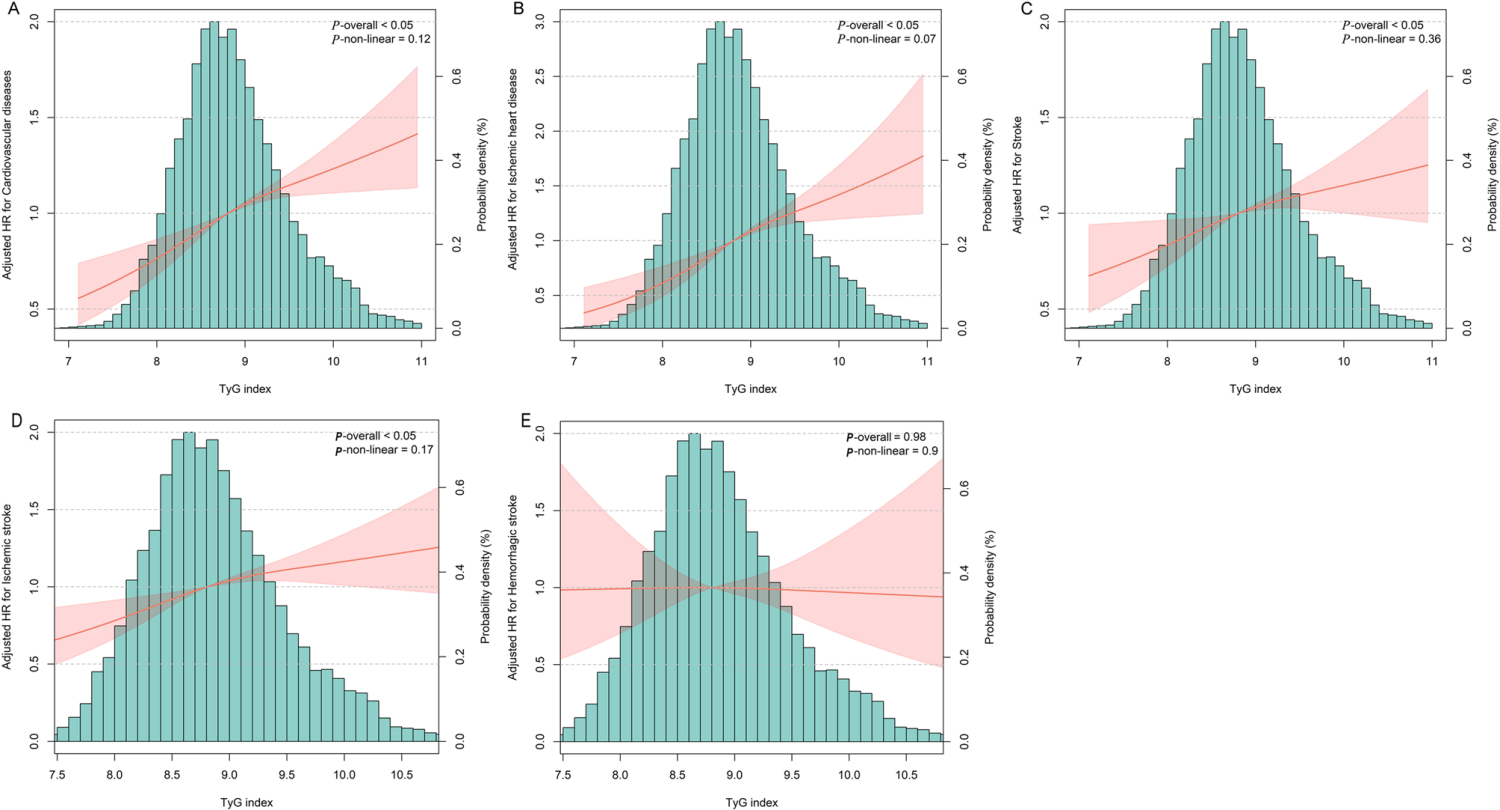
Associations of TyG index with the risk of cardiovascular diseases and its subtypes using restricted cubic spline regression models (Model 3) Panel **A**: Cardiovascular diseases; Panel **B**: Ischemic heart disease; Panel **C**: Stroke; Panel **D**: Ischemic stroke; Panel **E**: Hemorrhagic stroke Point estimates (solid lines) and 95% confidence intervals (dashed lines) were obtained from restricted cubic spline regression models adjusted for covariates in Model 3

During a mean (SD) follow-up of 14.19 (3.72) years, there were 2,537 new cases of CVD events among all participants, corresponding to an incidence rate of 9.73 events per 1,000 person-years. The cumulative incidence of CVD according to the TyG index quartile groups is depicted in Fig. 2 (log-rank test, *P* < 0.05). In Cox proportional hazards regression multivariable analysis, compared with the first TyG index quartile group (Q1, TyG index < 8.45), the multivariable-adjusted HRs were 1.18 (95% CI, 1.05–1.34) for the Q2 (TyG index 8.45–8.81), 1.29 (95% CI, 1.15–1.46) for the Q3 (TyG index 8.81–9.23), and 1.45 (95% CI, 1.28–1.63) for the Q4 (TyG index ≥ 9.23) (Table 2). For every 1-unit increase in TyG index, the multivariable adjusted HR was 1.20 (95% CI, 1.16–1.24) for CVD (Table 2). Adjusted PAR (95% CI) for incident CVD associated with a high TyG index was 7.04 (4.50–9.59) (Table 2). The cubic spline models (Fig. 3) showed a linear dose–response association between TyG index levels and the risk of incident CVD (*P*-non-linear = 0.12).

**Table 2:** Association between the level of TyG index and the risk of cardiovascular diseases and its subtypes in patients with prediabetes

	HR (95% CI)				
	Q1 (*N* = 4584)	Q2 (*N* = 4598)	Q3 (*N* = 4591)	Q4 (*N* = 4591)	Per 1-unit increase
Cardiovascular diseases, n (%)	466 (10.17)	604 (13.14)	694 (15.12)	773 (16.84)	
Incidence density (per 1000 persons-years)	7.07	9.28	10.76	11.87	
Model 1	Reference	1.30 (1.16–1.47)	1.51 (1.35–1.70)	1.77 (1.58–1.99)	1.20 (1.16–1.24)
Model 2		1.30 (1.15–1.46)	1.51 (1.35–1.70)	1.77 (1.58–1.99)	1.20 (1.16–1.24)
Model 3		1.18 (1.05–1.34)	1.29 (1.15–1.46)	1.45 (1.28–1.63)	1.12 (1.08–1.17)
Population attributable risk, %	-	2.31 (0.65–4.28)	4.20 (2.22–6.50)	7.04 (4.50–9.59)	
Ischemic heart disease, n (%)	135 (2.95)	201 (4.37)	241 (5.25)	316 (6.88)	
Incidence density (per 1000 persons-years)	1.99	2.99	3.60	4.68	
Model 1	Reference	1.49 (1.20–1.86)	1.79 (1.45–2.21)	2.45 (2.01-3.00)	1.33 (1.25–1.41)
Model 2		1.48 (1.19–1.84)	1.79 (1.45–2.21)	2.46 (2.01–3.02)	1.33 (1.25–1.41)
Model 3		1.35 (1.08–1.68)	1.50 (1.21–1.86)	1.97 (1.59–2.43)	1.24 (1.16–1.32)
Population attributable risk, %	-	1.51 (0.35–2.89)	2.56 (1.09–4.32)	6.26 (3.90–8.96)	
Stroke, n (%)	346 (7.55)	425 (9.24)	483 (10.52)	501 (10.91)	
Incidence density (per 1000 persons-years)	5.19	6.42	7.34	7.49	
Model 1	Reference	1.23 (1.07–1.42)	1.40 (1.22–1.61)	1.52 (1.33–1.75)	1.15 (1.10–1.20)
Model 2		1.22 (1.06–1.40)	1.40 (1.22–1.61)	1.52 (1.33–1.75)	1.15 (1.10–1.20)
Model 3		1.12 (0.97–1.29)	1.21 (1.05–1.40)	1.26 (1.09–1.45)	1.08 (1.03–1.13)
Population attributable risk, %	-	1.10 (−0.28-2.61)	2.16 (0.52–4.04)	2.76 (0.97–4.68)	

Q1: TyG index < 8.45, Q2: TyG index 8.45–8.81, Q3: TyG index 8.81–9.23, Q4: TyG index ≥ 9.23

*Abbreviation: TyG* triglyceride glucose, *hs-CRP* high-sensitivity C-reactive protein, *CVD* cardiovascular diseases, *HR* hazard ratio, *CI* confidence interval

Model 1 adjusted for age and sex

Model 2 adjusted for variables in model 1 and current smoking, current drinking, physical activity, education, snoring, and CVD family history

Model 3 adjusted for variables in model 2 and body mass index, hypertension, total cholesterol, low density lipoprotein cholesterol, high density lipoprotein cholesterol, log hs-CRP, antihypertensive and lipid-lowering drugs

### Subgroup analyses

**Fig. 4:**
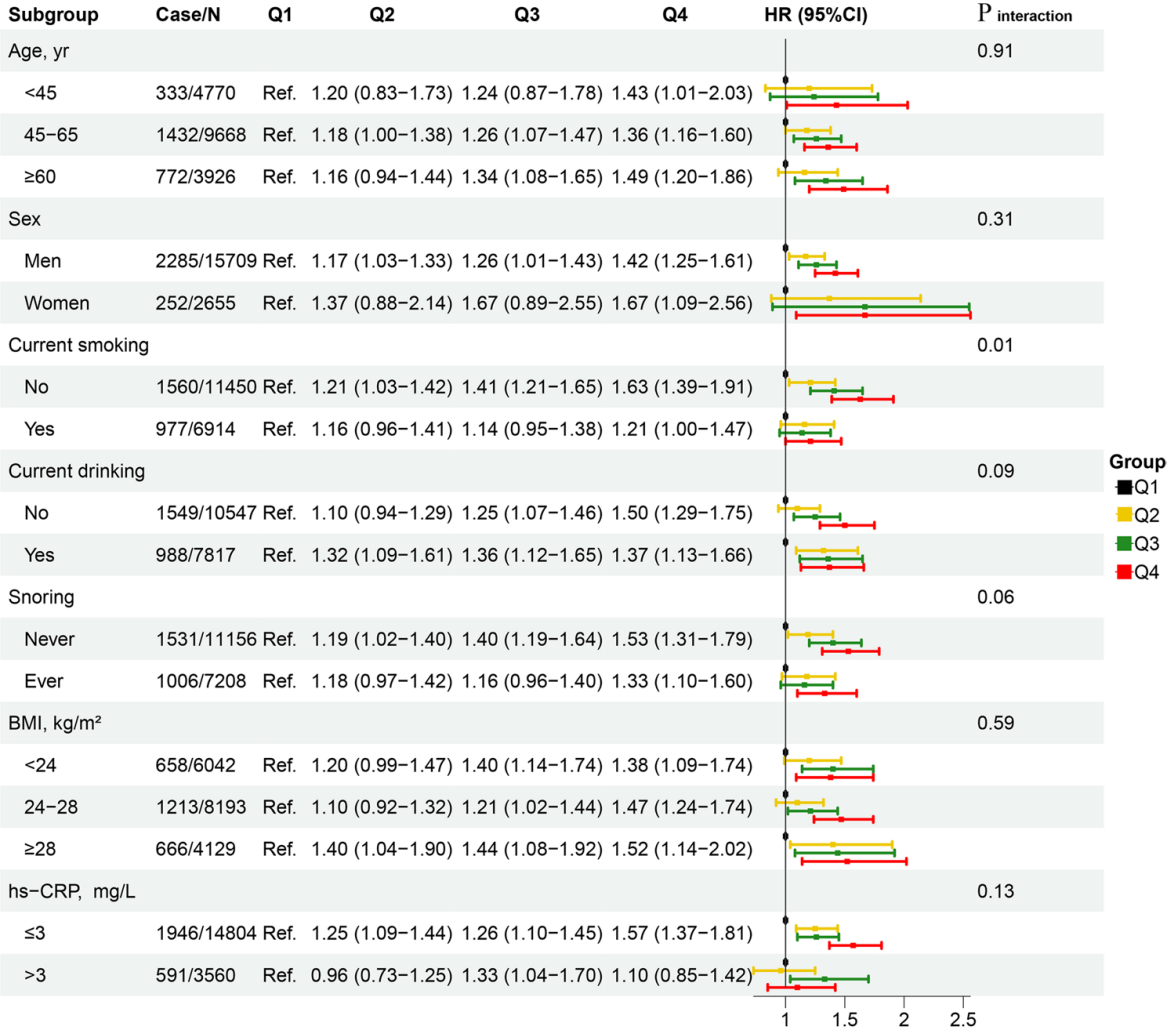
Forest plot of multivariate-adjusted hazard ratios for the association between TyG index categories and cardiovascular diseases across different stratifications Model adjusted for age, sex, current smoking, current drinking, physical activity, education, snoring, CVD family history, BMI, hypertension, total cholesterol, low density lipoprotein cholesterol, high density lipoprotein cholesterol, log hs-CRP, antihypertensive, and lipid-lowering drugs. For the stratified analyses, the stratification factor was not included in the model

Subgroup analyses of TyG index levels and CVD risk among patients with prediabetes are shown in Fig. 4. There were significant interactions between the TyG index and smoking status, drinking status, and snoring (all *P*_interaction_<0.10). Except for the subgroup with hs-CRP > 3 mg/L, the association between TyG index and CVD risk was significant and consistent across all other subgroups. In the hs-CRP > 3 mg/L subgroup, however, the association was weaker and not statistically significant.

### Sensitivity analyses

A series of sensitivity analyses were performed to examine the robustness of the association between TyG index levels and CVD risk in prediabetes patients. The results of the sensitivity analyses were consistent with the main findings. Furthermore, based on Model 3, we calculated the E-Value for the association between the fourth quartile of the TyG index and CVD, which was 2.26. This suggests that any confounding factor associated with CVD would need to have a HR of at least 2.26 to reduce the observed HR for CVD events to below 1. Meanwhile, the competing risks analysis using the Fine–Gray model showed a trend similar to that of the original analysis (Supplemental Table 1).

### Prediction model

The addition of TyG index to the China-PAR model in the overall population resulted in a higher predictive performance for the risk of CVD compared with the addition of TG or FBG. The C-index for predicting the occurrence of CVD was 68.1%, which was a 0.4% improvement compared with the China-PAR model alone (C-index = 67.7%). The continuous NRI was 0.102 (95% CI 0.076–0.128) and the IDI was 0.001 (95% CI 0.001–0.003) (Supplemental Table 2).

### Secondary outcomes

During the follow-up period, there were 893 incident cases of ischemic heart disease, 1,755 cases of stroke, 1,548 cases of ischemic stroke, and 281 cases of hemorrhagic stroke. A higher TyG index was significantly associated with increased risks of ischemic heart disease (HR per 1-unit increase: 1.24; 95% CI: 1.16–1.32), stroke (HR: 1.08; 95% CI: 1.03–1.13), and ischemic stroke (HR: 1.11; 95% CI: 1.05–1.16), but not with hemorrhagic stroke (HR: 0.98; 95% CI: 0.87–1.09) among prediabetic patients (Table 2 and Supplemental Table 2). All associations followed a linear trend (P for non-linearity > 0.05), except for hemorrhagic stroke, which showed no significant association (P for overall association = 0.98) (Fig. 3).

## Discussion

### Principal findings

In this large, community-based, prospective cohort study, we demonstrated that a higher TyG index, either as a continuous or categorical variable, was significantly associated with an increased risk of CVD among patients with prediabetes, particularly for ischemic heart disease. The association between the TyG index and CVD risk in prediabetic patients was consistent across subgroups by age, sex, BMI, smoking, drinking, and snoring status. The association was stronger in women, older adults, those with obesity, lower systemic inflammation (hs-CRP ≤ 3 mg/L), and non-smokers, non-drinkers, and non-snorers. The estimated PAR further suggested that 7.04% of incident CVD events could have been avoided if all prediabetes participants had maintained a low TyG index. In addition, the TyG index significantly improved risk prediction when added to conventional models, supporting its potential clinical utility.

### Comparison with other studies

Observational prospective studies on the associations between TyG index and CVD risk among patients with prediabetes are lacking; therefore, no direct comparison was possible. Previous cross-sectional research by Liu et al. found an OR of 1.65 (95% CI, 1.20–2.25) for CVD associated with each one-point increase in the TyG index, among 4,340 prediabetic or diabetic patients younger than 65 years [22]. Similarly, an analysis of 6,488 patients with diabetes or prediabetes from the 2006–2017 National Health and Nutrition Examination Survey (NHANES) data yielded similar results [34]. These studies, however, have mainly focused on populations that include both prediabetes and diabetes patients. According to the report by Liu et al., there were differences in the dose-dependent association between the TyG index and CVD in prediabetes versus diabetes patients, with a positive association observed in prediabetes patients and a U-shaped association in diabetes patients. This suggests that to achieve desired health outcomes, different types of intervention measures and health goals might be required for each group. By focusing solely on prediabetic patients within a prospective cohort, our study is the first to demonstrate that an elevated TyG index is already associated with an increased risk of incident CVD at the prediabetes stage. This is crucial for the early identification of high-risk individuals for CVD, especially those who have not been diagnosed with diabetes but already exhibit disturbances in blood glucose and lipid metabolism. Furthermore, we extended our analysis beyond association by evaluating the predictive utility of the TyG index. Incorporating TyG into conventional risk models significantly improved discrimination and reclassification indices, supporting its potential as a simple, accessible marker for early cardiovascular risk stratification in prediabetic populations.

The TyG index is more strongly associated with ischemic heart disease than with stroke in those with prediabetes. Our results were broadly similar to those reported in the general population [18, 35]. Hong et al. used longitudinal data from the National Health Information Database (NHID) and found that compared to individuals with the lowest TyG index, those in the highest quartile had a 1.31-fold increased risk of myocardial infarction and a 1.26-fold increased risk of stroke [36]. Notably, the difference in the strength of association was even more pronounced among prediabetes patients.

In a study on the general population, Hong et al. discovered that the TyG index levels were more strongly associated with CVD risk in men, patients under the age of 65, and alcohol drinkers [36]. Furthermore, another study observed that among diabetic patients, the association between the TyG index and CVD was more pronounced in women and in those who do not smoke or drink alcohol [37]. However, no research has yet explored the association between the TyG index and CVD risk across different ages, sexes, BMI, inflammation levels, and lifestyles in prediabetic patients. Our study identified statistically significant interactions between the TyG index and smoking, drinking, and snoring status. These lifestyle factors may have a synergistic effect with the metabolic disturbances indicated by a high TyG index. Specifically, smoking, drinking, and snoring are associated with pro-inflammatory and pro-thrombotic states [38, 39]which could further exacerbate the metabolic dysfunction linked to TyG and amplify CVD risk. We also found that the association between the TyG index and CVD was weaker and not statistically significant among individuals with hs-CRP > 3 mg/L. This attenuation may reflect a ceiling effect, where patients with elevated baseline inflammation already have a heightened CVD risk, reducing the incremental impact of the TyG index. Additionally, the relatively small sample size in this subgroup may have limited statistical power to detect a significant association.

### Potential mechanisms

The mechanism by which TyG indices are associated with increased CVD remains unclear. However, insulin resistance may hold a key position between the association of TyG with CVD. Insulin resistance, a pathophysiological condition, diminishes cellular responsiveness to insulin, thereby initiating hyperglycemia [40]. This hyperglycemic state exacerbates glycosylation processes and reduces nitric oxide bioavailability, impairing endothelium-dependent vasodilation and promoting endothelial dysfunction [41–43]. Moreover, hyperglycemia induces inflammatory responses that stimulate smooth muscle cell proliferation and collagen deposition, resulting in vascular rigidity and impaired cardiac function [15, 44–46]. Moreover, insulin resistance is closely associated with enhanced oxidative stress, which contributes to vascular endothelial dysfunction and accelerates the progression of atherosclerosis, further elevating the risk of CVD [47, 48]. Our study found that the TyG index was significantly associated with ischemic stroke, but not with hemorrhagic stroke, further emphasizing the role of metabolic dysfunction and atherosclerosis in the development of cerebrovascular events. In addition, hyperinsulinemia -a hallmark of insulin resistance- activates the sympathetic nervous system, increasing the secretion of catecholamines such as adrenaline and norepinephrine. This activation establishes a feedback loop of neural and humoral overactivity, culminating in a rise in cardiac output and increased peripheral vascular resistance [49, 50]. These combined effects of insulin resistance further contribute to the progression of atherosclerosis, vascular rigidity, and ultimately the increased risk of CVD.

### Strengths and limitations

Strengths of this study include its prospective design, long-term follow-up, a large-scale cohort of patients with prediabetes, and the use of multiple sensitivity analyses to verify the stability of the results. Moreover, the entire study population was covered by the Municipal Social Insurance Institution, hospital discharge registers, and biennial medical examinations, which enabled us to track outcome events for all participants.

Despite these strengths, it is important to acknowledge the limitations of the present study. First, the diagnosis of prediabetes was determined based on a single FBG measurement [51, 52]rather than using oral glucose tolerance testing or measuring hemoglobin A1c, which could lead to an underestimation in identifying prediabetes. Future studies that include all of these tests are needed to verify the results. Second, the observational study design made it impossible to establish cause-effect association among the investigated variables. Further basic research and Mendelian randomization studies are necessary to confirm these findings. Third, the study population was limited to occupational individuals from northern China, which may limit the generalizability of the results to other populations and regions. Fourth, although we estimated the PAR to quantify the potential contribution of elevated TyG index to CVD burden, this calculation inherently assumes a causal relationship between exposure and outcome. Given the non-randomized observational design of our study, causality cannot be firmly established, and unmeasured confounding may still exist. Therefore, the PAR estimate should be interpreted as a hypothetical approximation rather than as a direct indication of preventable risk. Fifth, we were unable to exclude participants with certain unmeasured comorbidities, such as thyroid dysfunction, which may influence lipid metabolism and cardiovascular risk. Data on thyroid function or related diagnoses were not available in our dataset, and thus we could not identify and exclude such individuals at baseline. Finally, although stratified and sensitivity analyses were conducted to assess the robustness of our findings, further validation in external cohorts and interventional studies is warranted.

## Conclusions

In summary, a higher TyG index was independently associated with a 45% increased risk of overall cardiovascular disease, a 97% increased risk of ischemic heart disease, and a 26% increased risk of stroke among patients with prediabetes. These findings hold significant public health and clinical implications, given the widespread prevalence of prediabetes and the elevated cardiovascular risk in this population.

## Supplementary Information

Supplementary Material 1.

## Data Availability

The data underlying this article will be shared on reasonable request to the corresponding authors.

## Abbreviations

CVD: Cardiovascular disease
TyG index: Triglyceride-glucose index
FBG: Fasting blood glucose
TG: Triglyceride
CABG: Coronary artery bypass grafting
PCI: Percutaneous coronary intervention
BMI: Body mass index
LDL-C: Low-density lipoprotein cholesterol
HDL-C: High-density lipoprotein-cholesterol
TC: Total cholesterol
hs-CRP: High-sensitivity C-reactive protein
HR: Hazard ratio
CI: Confidence interval
PAR: Population-attributable risk
