# Supplementary material for: Association of triglyceride-glucose index with risk of cardiovascular disease among patients with prediabetes: population based prospective cohort study
Source: BMC Cardiovasc Disord. 2025 Sep 29;25:685. doi: 10.1186/s12872-025-05112-w (PMC12481991; doi:10.1186/s12872-025-05112-w)
Supplement: Supplementary file 1 — Supplementary Material 1. [file 12872_2025_5112_MOESM1_ESM.docx]

**Supplemental Table 1.** Sensitivity analysis for the association between the TyG index level and the risk of cardiovascular diseases in prediabetic patients

|  | HR (95% CI) | | | | |
| --- | --- | --- | --- | --- | --- |
|  | Q1 | Q2 | Q3 | Q4 | Per 1-unit increase |
| Model 1^a^ | Reference | 1.20 (1.06-1.36) | 1.30 (1.15-1.47) | 1.45 (1.28-1.64) | 1.14 (1.08-1.17) |
| Model 2^b^ |  | 1.18 (1.03-1.35) | 1.31 (1.15-1.49) | 1.49 (1.31-1.70) | 1.14 (1.09-1.19) |
| Model 3^a^ |  | 1.17 (1.03-1.32) | 1.26 (1.12-1.43) | 1.43 (1.27-1.62) | 1.12 (1.08-1.17) |
| Model 4^a^ |  | 1.64 (1.28-2.01) | 1.90 (1.57-2.28) | 2.26 (1.88-2.64) | 1.49 (1.37-1.62) |
| Model 5^a^ |  | 1.18 (1.05-1.34) | 1.30 (1.15-1.47) | 1.46 (1.30-1.66) | 1.13 (1.09-1.17) |
| Model 6^c^ |  | 1.04 (0.92-1.17) | 1.11 (0.99-1.25) | 1.15 (1.02-1.30) | 1.05 (1.01-1.09) |
| Model 7^d^ |  | 1.16 (1.03-1.32) | 1.24 (1.10-1.40) | 1.34 (1.18-1.53) | 1.10 (1.05-1.14) |
| Model 8^a^ |  | 1.23 (1.08-1.40) | 1.35 (1.18-1.53) | 1.53 (1.35-1.74) | 1.14 (1.10-1.19) |
| Model 9^a^ |  | 1.17 (1.021.35) | 1.30 (1.13-1.50) | 1.57 (1.36-1.81) | 1.16 (1.11-1.21) |
| Q1: TyG index <8.45, Q2: TyG index 8.45-8.81, Q3: TyG index 8.81-9.23, Q4: TyG index ≥9.23;  Model 1 excluding new-onset cardiovascular diseases were followed up within 1 years (N=18,225).  Model 2 excluding participants with antihypertensive and lipid-lowering drugs at baseline (N=16,282).  Model 3 Using the complete dataset without imputation.  Model 4 E value.  Model 5 Competing risk analysis.  Model 6: Using TyG index data from the most recent clinical examination before the end of follow-up as the exposure variable.  Model 7: Further adjusted for TyG index data from the most recent clinical examination before the end of follow-up, based on the original model.  Model 8: Excluding participants who developed hemorrhagic stroke during follow-up (N = 18,083).  Model 9: Excluding participants who developed diabetes during follow-up (N = 12,395).  ^a^ Model adjusted for age, sex, current smoking, current drinking, physical activity, education, snoring, CVD family history, body mass index, hypertension, total cholesterol, low density lipoprotein cholesterol, high density lipoprotein cholesterol, log hs-CRP, antihypertensive, and lipid-lowering drugs.  ^b^ Model adjusted for age, sex, current smoking, current drinking, physical activity, education, snoring, CVD family history, body mass index, hypertension, total cholesterol, low density lipoprotein cholesterol, high density lipoprotein cholesterol, and log hs-CRP.  ^c^ Model adjusted for TyG index (data from baseline examination), age, sex, current smoking, current drinking, physical activity, education, snoring, CVD family history, body mass index, hypertension, total cholesterol, low density lipoprotein cholesterol, high density lipoprotein cholesterol, log hs-CRP, antihypertensive, and lipid-lowering drugs.  ^d^ Model adjusted for TyG index (data from the most recent clinical examination before the end of follow-up), age, sex, current smoking, current drinking, physical activity, education, snoring, CVD family history, body mass index, hypertension, total cholesterol, low density lipoprotein cholesterol, high density lipoprotein cholesterol, log hs-CRP, antihypertensive, and lipid-lowering drugs.  Abbreviation: TyG, Triglyceride Glucose and hs-CRP, high-sensitivity C-reactive protein; CVD, cardiovascular diseases; Ref, reference; HR, hazard ratio and CI, confidence interval. | | | | | |

**Supplemental Table 2.** C-index，NRI and IDI of different indicators for cardiovascular diseases in prediabetic patients

| Predictor | C-Index (95%CI) | Continuous NRI (95%CI) | Absolute IDI (95%CI) |
| --- | --- | --- | --- |
| China-PAR model | 0.677 (0.667-0.687) | Reference | Reference |
| China-PAR model + TG | 0.678 (0.669-0.689) | 0.090 (0.031-0.092) | 0.001 (0.001-0.003) |
| China-PAR model + FBG | 0.678 (0.668-0.688) | 0.067 (0.041-0.132) | 0.001 (0.001-0.003) |
| China-PAR model + TyG index | 0.681 (0.671-0.691) | 0.102 (0.076-0.128) | 0.001 (0.001-0.003) |
| Abbreviation: TG, Triacylglycerol; FBG, Fasting blood glucose; TyG, Triglyceride Glucose; China-PAR model, Prediction Model for Atherosclerotic Cardiovascular Disease Risk in China; IDI. the integrated discrimination index; NRI the net reclassification index and Cl, confidence interval. | | | |

**Supplemental Table 3.** Association between the level of TyG index and the risk of ischemic stroke and hemorrhagic stroke in patients with prediabetes

|  | HR (95% CI) | | | | |
| --- | --- | --- | --- | --- | --- |
|  | Q1  (N=4584) | Q2  (N=4598) | Q3  (N=4591) | Q4  (N=4591) | Per 1- unit increase |
| **Ischemic stroke**, n (%) | 291 (6.35) | 370 (8.05) | 436 (9.50) | 451 (9.82) |  |
| Incidence density^a^ | 4.35 | 5.56 | 6.59 | 6.70 |  |
| Model 1 | Reference | 1.27 (1.09-1.49) | 1.51 (1.30-1.75) | 1.63 (1.41-1.89) | 1.17 (1.12-1.23) |
| Model 2 |  | 1.27 (1.09-1.48) | 1.51 (1.30-1.76) | 1.63 (1.41-1.89) | 1.17 (1.12-1.23) |
| Model 3 |  | 1.17 (1.00-1.36) | 1.31 (1.12-1.53) | 1.36 (1.16-1.58) | 1.11 (1.05-1.16) |
| **Hemorrhagic stroke**, n (%) | 67 (1.46) | 69 (1.50) | 74 (1.61) | 71 (1.55) |  |
| Incidence density^a^ | 0.98 | 1.01 | 1.09 | 1.03 |  |
| Model 1 | Reference | 1.02 (0.73-1.43) | 1.09 (0.78-1.52) | 1.11 (0.79-1.55) | 1.04 (0.93-1.15) |
| Model 2 |  | 1.01 (0.72-1.41) | 1.09 (0.78-1.52) | 1.10 (0.79-1.54) | 1.04 (0.93-1.15) |
| Model 3 |  | 0.93 (0.66-1.30) | 0.96 (0.68-1.35) | 0.91 (0.64-1.30) | 0.98 (0.87-1.09) |
| Q1: TyG index <8.45, Q2: TyG index 8.45-8.81, Q3: TyG index 8.81-9.23, Q4: TyG index ≥9.23; Model 1 adjusted for age and sex. Model 2 adjusted for variables in model 1 and current smoking, current drinking, physical activity, education, snoring, and CVD family history. Model 3 adjusted for variables in model 2 and body mass index, hypertension, total cholesterol, low density lipoprotein cholesterol, high density lipoprotein cholesterol, log hs-CRP, antihypertensive and lipid-lowering drugs; Abbreviation: TyG, triglyceride glucose; hs-CRP, high-sensitivity C-reactive protein; CVD, cardiovascular diseases; HR, hazard ratio; CI, confidence interval; ^a^: per 1000 persons-years | | | | | |
